# Yoga training enhances elastic biomechanics of trapezius and hamstrings: a quantitative SWE assessment

**DOI:** 10.3389/fphys.2025.1671051

**Published:** 2026-01-13

**Authors:** Jing Liu, Lin Yang

**Affiliations:** Department of Ultrasound, Santai People’s Hospital, Mianyang, China

**Keywords:** elasticity assessment, flexibility training, muscle stiffness, neuromuscular coordination, shear wave velocity, ultrasound biomechanics

## Abstract

**Aim:**

To investigate the association between long-term Yoga practice and changes in trapezius and hamstring biomechanics using shear-wave elastography (SWE), and to identify reliable measurement conditions.

**Methods:**

Eighty-one healthy women were assigned to a Yoga group (n = 51) or non-Yoga control group (n = 30). The mean Young’s modulus (E_mean_) of the trapezius, biceps femoris long head, and semitendinosus was assessed at baseline, 1, 3, and 6 months under standardized postures (neutral, flexed, extended, and 90° knee flexion) and imaging planes (transverse/longitudinal) using a Siemens ACUSON Redwood system.

**Results:**

Eighty-one healthy adult women were enrolled (Yoga: n = 51; non-Yoga: n = 30), with no significant baseline differences between groups (all p > 0.05). Muscle thickness did not differ between groups at any time point for any muscle (all p > 0.05). For the trapezius, significant main effects of position (p < 0.001) and time (p = 0.014), as well as a significant group × time interaction (p < 0.001), were observed, with a progressive reduction in Emean in the Yoga group only. In the hamstring muscles, significant group × time interactions were detected for both the biceps femoris long head (longitudinal: F (2.66, 210.24) = 12.11, p < 0.001; transverse: F (2.42, 191.04) = 9.19, p < 0.001) and the semitendinosus (longitudinal: F (2.05, 161.87) = 9.93, p < 0.001; transverse: F (2.47, 195.00) = 5.21, p = 0.002). Longitudinal measurements consistently showed greater sensitivity to temporal changes than transverse measurements, and weak inverse correlations were observed between cumulative yoga training duration and muscle elasticity (all p < 0.05).

**Conclusion:**

Six months of regular Yoga was associated with improved muscle elasticity, while SWE provides a robust and quantitative assessment of posture-dependent biomechanical adaptations.

## 1 Introduction

Yoga is a widely practiced mind-body exercise with documented benefits for flexibility, muscle function, and overall wellbeing, especially in women (Malone and Gloyer, 2013; Bucea-Manea-Tonis and Paun, 2024). Regular yoga practice has been shown to improve balance, range of motion, and muscular strength in healthy female populations (e.g., a 10-week hatha yoga program improved flexibility and core strength in young women) (Jeitler et al., 2020; Csala et al., 2021; Xu et al., 2022; Krishnamurthy, 2023). Such findings provide a strong rationale to investigate yoga’s biomechanical effects on muscle tissue. In particular, healthy women often experience muscle tightness (e.g., hamstrings) or postural strain (e.g., trapezius) that yoga’s stretching and strengthening components might alleviate. High muscle stiffness in the hamstrings is linked to reduced flexibility and injury risk (Rodoplu et al., 2025), and excessive trapezius stiffness is associated with neck shoulder pain in women, as demonstrated by shear-wave elastography studies showing increased upper trapezius stiffness in female patients with neck–shoulder pain (Hao et al., 2023). Yet, the impact of long-term yoga training on objective muscle elasticity (stiffness) remains poorly understood. This is a notable gap, given that female muscle properties can differ from males for instance, shear-wave elastography studies report lower upper trapezius stiffness in women compared to men (Kozinc and Sarabon, 2020) and show that hormonal cycles can modulate muscle stiffness in young women (with implications for injury risk) (Khowailed et al., 2022). These sex-specific factors underscore the importance of focusing on women when assessing interventions aimed at improving muscle mechanical properties.

In modern medicine, there is a growing demand for the scientific evaluation of complementary and alternative therapies, and the validity and efficacy of Yoga as an alternative intervention have attracted increasing attention (Lysogorskaia et al., 2023; Antoniou and Georgiadis, 2024) Advanced diagnostic tools such as electromyography, dynamic posture and balance assessments, and three-dimensional motion capture are now employed to quantitatively evaluate Yoga’s biomechanical effects on specific muscle groups (Salem et al., 2013; Devaraju et al., 2019; Mullerpatan et al., 2020). These studies collectively support Yoga as a promising non-pharmacological approach for improving musculoskeletal and mental health outcomes (Wieland et al., 2022; Metri et al., 2023).

Shear-wave elastography (SWE) offers a non-invasive, quantitative means to evaluate muscle elasticity *in vivo* by measuring shear wave propagation speed through tissue (Devaraju et al., 2019; Tagliafico, 2020; Romero-Morales et al., 2021; Sarto et al., 2021; Venkatesan et al., 2022; Pan, 2024). Unlike conventional ultrasound that visualizes morphology, SWE directly measures intrinsic tissue stiffness independent of patient effort. SWE-derived parameters like shear-wave velocity and Young’s modulus serve as indicators of muscle stiffness and function (Taljanovic et al., 2017; Licen and Kozinc, 2022). The technique has proven reliable for longitudinal muscle assessment, for example, SWE shows good repeatability in trapezius stiffness measurementsand can sensitively detect changes in muscle mechanics under different conditions (Kozinc and Sarabon, 2020). Importantly, SWE is well-suited for tracking subtle adaptations over time, often capturing improvements in muscle pliability before gross structural changes occur (Alfuraih et al., 2019; Hao et al., 2023). It has been validated in various musculoskeletal applications and increasingly applied to monitor exercise-induced changes in muscle properties. Recent studies in biomechanics highlight SWE’s utility in female cohorts and training contexts. For instance, Khowailed et al. (2022) used SWE in healthy women to show menstrual-phase differences in calf muscle stiffness, and Kozinc and Sarabon (2020) confirmed SWE’s reliability for trapezius stiffness while noting sex-based differences. A case-control study revealed abnormally reduced SWV in the hamstrings of patients with idiopathic inflammatory disease, correlating with muscle weakness and edema signs (Alfuraih et al., 2019). Furthermore, flexibility-focused interventions are known to reduce muscle stiffness: a meta-analysis found that 3–12 weeks of static stretching led to a moderate decrease in musculotendinous stiffness (Takeuchi et al., 2023). This evidence suggests that a prolonged yoga regimen which entails sustained stretching and muscle engagement could similarly enhance muscle elasticity, yet this hypothesis has not been rigorously tested with direct elasticity measures.

In light of these considerations, the present study was designed to examine the association between participation in a 6-month yoga training program and longitudinal changes in muscle stiffness in healthy adult women. We specifically targeted the upper trapezius and hamstring (biceps femoris and semitendinosus) muscles, given their functional importance and susceptibility to tightness, and employed SWE to quantify muscle elastic properties. A controlled observational design with a 6-month follow-up period was implemented, with muscle stiffness evaluated in both a regular yoga-practicing group and a non-exercising control group at multiple time points. Measurements were performed in standardized postures (including transverse-plane positions) to ensure consistent, angle-specific assessment of elasticity. We hypothesized that participation in regular yoga practice would be associated with lower trapezius and hamstring muscle stiffness (i.e., higher elasticity) over time compared with no yoga practice. By leveraging SWE’s sensitivity in a three-way repeated-measures design (group × position × time), this study aims to fill the knowledge gap on how long-term yoga practice is associated with variations in muscle Emean in women. Ultimately, our goal is to clarify whether yoga practice is associated with favorable changes in muscle elasticity *in vivo*, thereby informing the link between mind–body exercise and musculoskeletal health.

## 2 Methods

### 2.1 Study design

This study employed a prospective cohort-controlled observational design to examine the association between participation in a 6-month yoga training program and changes in the E_mean_ of the trapezius and hamstring muscles in healthy adult women. A total of 81 healthy adult women were recruited locally and assigned to two groups based on self-selection and their willingness to participate in yoga training during the study period: 51 participants who voluntarily undertook yoga training after enrollment (Yoga group) and 30 participants who did not practice yoga and continued their usual daily activities (Non-Yoga group). Basic clinical data, including age, height, body weight, and body mass index (BMI), were collected for both groups. The study protocol received approval from the Ethics Committee of Santai People’s Hospital (approval number: 2023011), complied with the ethical principles outlined in the *Declaration of Helsinki*, and rigorously protected participant rights and data privacy.

Inclusion criteria were: 1. healthy women aged between 26 and 50 years; 2. absence of significant underlying medical conditions, relevant surgical history, or long-term medication use; and 3. willingness to either participate in a yoga training program (Yoga group) or refrain from yoga practice (Non-Yoga group) during the 6-month study period.

Exclusion criteria comprised: 1. history of severe illness or significant injury; 2. anticipated poor compliance with the training protocol; and 3. presence of concomitant joint disorders or neuromuscular diseases.

### 2.2 Instruments and examination methods

**FIGURE 1:**
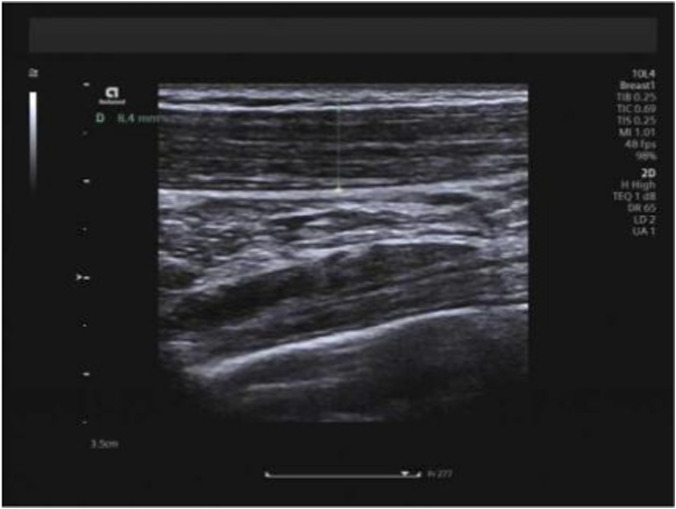
Measurement of trapezius thickness.

All examinations were performed using a Siemens ACUSON Redwood color Doppler ultrasound system (Siemens Healthineers, Erlangen, Germany) equipped with a 10L4 high-frequency linear array transducer (frequency range: 2.9–9.9 MHz). For trapezius muscle assessment, participants were positioned sitting upright with their upper limbs relaxed and hanging naturally at their sides, shoulders relaxed, and legs separated shoulder-width apart. The transducer was placed transversely over the right upper trapezius muscle, specifically targeting the midpoint of the line connecting the muscle’s inferior origin and insertion (determined by trisecting this line and selecting the central segment as the measurement site) to measure muscle thickness (Figure 1). Muscle thickness was defined as the perpendicular distance between the superficial and deep fascial borders on B-mode ultrasound images, and the mean value of three consecutive measurements was recorded. Trapezius measurements were acquired in three distinct head positions: neutral, maximal forward flexion, and maximal extension. All shear-wave elastography measurements of the trapezius, biceps femoris long head, and semitendinosus muscles were consistently performed on the right side of the body for all participants and at all time points to ensure side-specific measurement consistency. Measurements were restricted to the right side of the body to ensure methodological consistency across participants, reduce examination time and participant burden, and minimize variability related to potential side-to-side differences in muscle mechanical properties. This unilateral measurement approach is commonly adopted in musculoskeletal ultrasound studies when bilateral comparison is not the primary objective.

**FIGURE 2:**
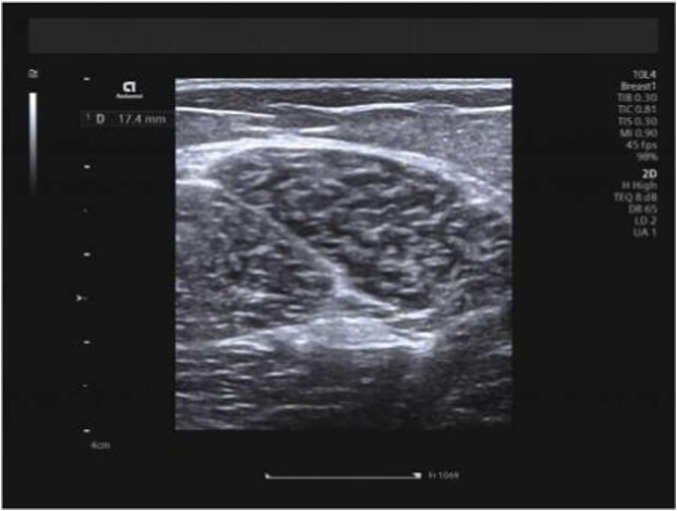
Measurement of biceps femoris long head thickness.

**FIGURE 3:**
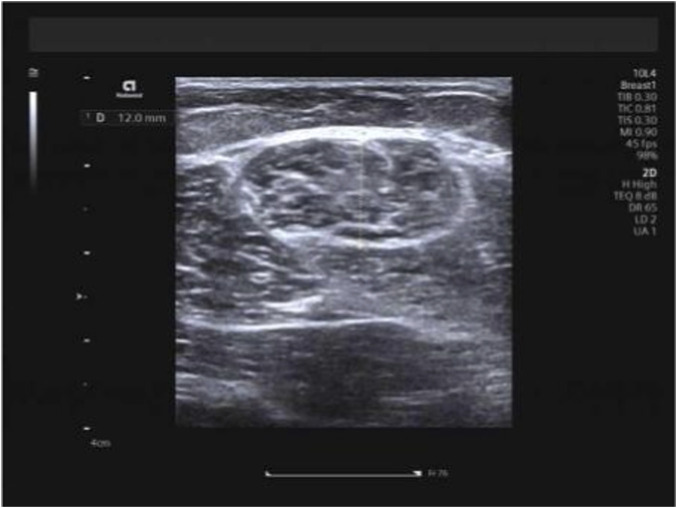
Measurement of semitendinosus thickness.

Hamstring muscles (specifically the long head of the biceps femoris and semitendinosus) were examined with participants in the prone position, legs extended naturally and body fully relaxed. The measurement site was standardized as the mid-belly region of each muscle, located approximately 15 cm distal to the ischial tuberosity along the longitudinal axis of the muscle fibers. Muscle thickness was measured on B-mode ultrasound images as the vertical distance between the superficial and deep fascia at this site, with the transducer placed transversely to the muscle fibers. Three consecutive measurements were taken and averaged to obtain the final value (Figures 2, 3). Hamstring measurements were obtained in two positions: the neutral position and the 90° knee-flexed position. During measurements performed at 90° knee flexion, the angle was maintained using a custom foam pad positioned beneath the lower leg to support the shank, ensuring a consistent 90° angle between the femur and tibia. The participant’s position was monitored throughout image acquisition to prevent unintended movement or muscle activation. In this study, measurement posture refers to the specific body position adopted during imaging (e.g., neutral, flexed, or extended), and measurement technique refers to the probe orientation or imaging plane (transverse or longitudinal) used during shear-wave elastography. Importantly, for all examined muscles—including the upper trapezius, biceps femoris long head, and semitendinosus—SWE measurements were performed in both longitudinal and transverse planes at the same anatomical locations. To ensure methodological consistency, the acquisition depth was standardized for each muscle group: 0.5–1.0 cm for the upper trapezius and 1.0–2.0 cm for the hamstring muscles (long head of the biceps femoris and semitendinosus), with minor adjustments made only to ensure that the ROI was positioned at the center of the muscle belly. A fixed 1.0 × 1.0 cm circular region of interest (ROI) was applied uniformly across all measurements. This dual-orientation approach was intentionally implemented to evaluate the consistency and direction-dependent characteristics of muscle stiffness, in accordance with previous recommendations.

**FIGURE 4:**
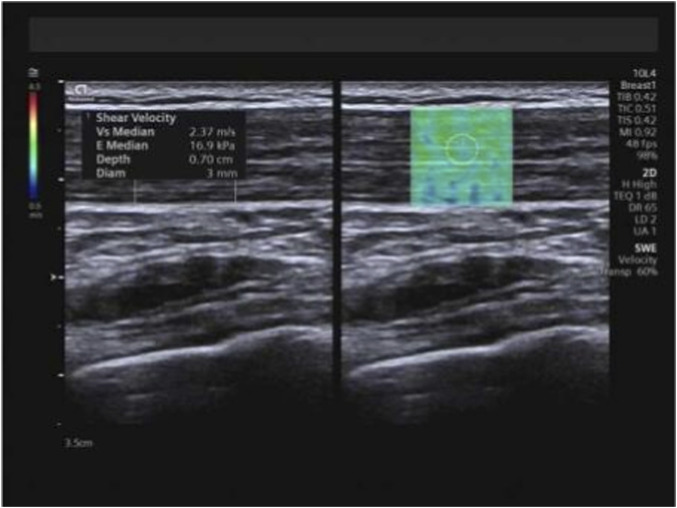
Measurement of E_mean_ and SWV_mean_ values for the trapezius. Note: E_mean_ denotes mean Young’s modulus; SWV_mean_ denotes mean shear wave velocity.

**FIGURE 5:**
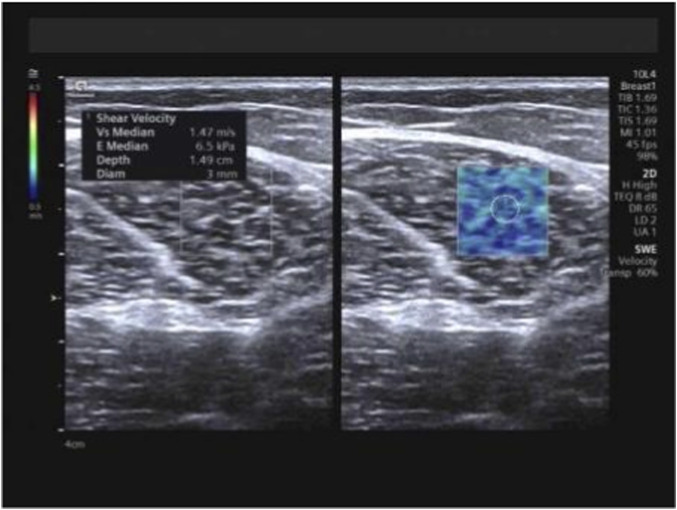
Measurement of E_mean_ and SWV_mean_ values for the long head of the biceps femoris. Note: E_mean_ denotes mean Young’s modulus; SWV_mean_ denotes mean shear wave velocity.

**FIGURE 6:**
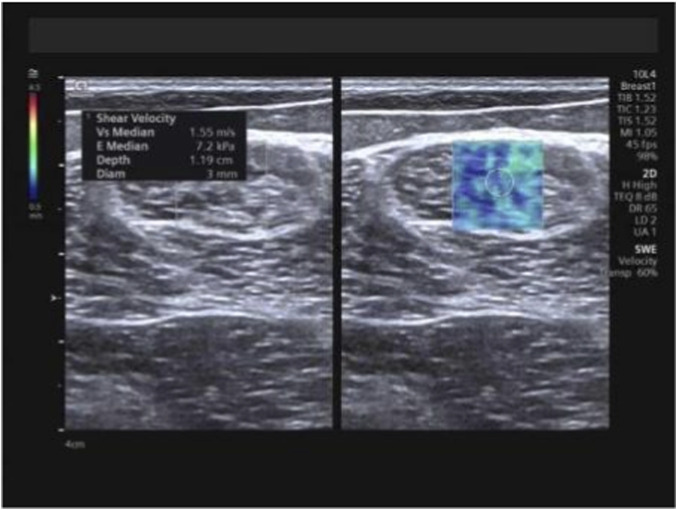
Measurement of E_mean_ and SWV_mean_ values for the semitendinosus. Note: E_mean_ denotes mean Young’s modulus; SWV_mean_ denotes mean shear wave velocity.

At the identical locations used for muscle thickness measurement, SWE examinations were performed pre-intervention and subsequently after 1 month, 3 months, and 6 months of training, utilizing the aforementioned measurement positions and techniques. Following conventional ultrasound imaging, the system was switched to SWE dual-display mode. The transducer was maintained perpendicular to the skin surface and carefully aligned parallel to the tendon/muscle fibers. The sample box was adjusted, and the image was frozen once stabilized. Using the system’s integrated measurement tools, E_mean_ and SWV_mean_ were measured, with the mean value derived from three consecutive measurements for each parameter (Figures 4–6).

### 2.3 Yoga training protocol

During the study period, participants in the Yoga group attended yoga training sessions three times per week for 6 months. Each session lasted approximately 60 min and followed a predefined structure to ensure consistency across participants. The training protocol primarily consisted of gentle Hatha-based yoga, including posture-based exercises (asanas) focusing on the neck–shoulder region and lower limb muscles, static stretching postures, and controlled transitions between postures. Each session included a standardized warm-up phase, a core asana practice phase, and a cool-down phase incorporating breathing exercises and relaxation. All sessions were supervised by certified yoga instructors, and attendance was recorded to monitor adherence. Attendance records indicated good adherence to the yoga training program. Participants attended a mean of 85% of the scheduled sessions over the 6-month intervention period (range: 70%–96%). Although minor individual adjustments were allowed for safety and comfort, the overall structure and training characteristics remained consistent throughout the study period. A detailed, tabulated description of the yoga intervention, including specific asanas, sequence, duration, breathing, relaxation, and progression, is provided in Table 1.

**TABLE 1:** Yoga training protocol.

Category	Description
Yoga style	Gentle hatha-based yoga
Program duration	6 months
Session frequency	3 sessions per week
Session duration	Approximately 60 min
Session structure	Warm-up, core asana practice, cool-down (breathing and relaxation)
Primary target regions	Neck–shoulder region and lower limb (hamstring) muscles
Core asanas	Tadasana, Adho Mukha Svanasana, Uttanasana, Anjaneyasana, Paschimottanasana, Setu Bandha Sarvangasana
Variants/Modifications	Knee flexion permitted during forward bends; reduced range of motion when required for comfort and safety
Sequence	Fixed sequence progressing from standing to seated and supine postures
Posture characteristics	Predominantly static postures with low-intensity transitional movements
Hold duration	Approximately 20–60 s per posture
Repetitions/Sets	1–3 repetitions per posture
Rest intervals	Approximately 10–20 s between repetitions
Breathing	Slow diaphragmatic breathing coordinated with posture execution; additional focused breathing during cool-down (∼5 min)
Relaxation	Supine relaxation (savasana), approximately 5–10 min
Progression	Overall structure fixed; gradual increase in hold duration and posture tolerance over the 6-month period
Adherence	Mean attendance of approximately 85% of scheduled sessions (range: 70%–96%) over the 6-month intervention period

### 2.4 Quality control

To ensure methodological consistency and minimize variability, all examinations were performed using the same color Doppler ultrasound system throughout the study. Strict adherence to standardized participant positioning protocols was maintained for all assessments. A single experienced physician performed all data acquisition and initial processing to eliminate operator-induced variability. Subsequently, the collected data and measurement outcomes underwent independent review and validation by three expert radiologists, each possessing over 10 years of specialized experience in MSK-US.

### 2.5 Statistical analysis

Data analysis was performed using SPSS software (version 25.0). Continuous variables conforming to a normal distribution were presented as mean ± standard deviation. Differences between the two groups (Yoga vs. non-Yoga) for these variables were assessed using independent samples *t*-tests. Comparisons across multiple groups involving normally distributed data were conducted using one-way analysis of variance (ANOVA). Continuous variables not adhering to a normal distribution were expressed as median (interquartile range, IQR), and inter-group differences were analyzed using the Mann-Whitney U test or the Kruskal-Wallis test. Categorical data were presented as proportions (percentages), and inter-group differences were evaluated using the Chi-square (χ^2^) test. Correlation analysis was performed to examine the relationship between cumulative yoga training duration and muscle biomechanical parameters. Cumulative yoga training duration was defined as the time elapsed since baseline assessment, expressed in months. A three-way repeated-measures analysis of variance (ANOVA) was conducted separately for the longitudinal and transverse planes, with group (Yoga vs. Non-Yoga) as the between-subject factor and position and time as within-subject factors, to examine differences in elasticity modulus (Emean) for each muscle. The sphericity assumption for within-subject factors was tested using Mauchly’s test. When sphericity was violated, Greenhouse–Geisser corrections were applied to adjust the degrees of freedom.

When a significant main effect or interaction was observed, *post hoc* pairwise comparisons with Bonferroni correction were performed. Effect sizes were reported using partial eta squared (ηp^2^) and interpreted as small (0.01–0.06), medium (0.06–0.14), or large (>0.14). All data were expressed as mean ± 95% confidence interval (95% CI). A *P*-value of less than 0.05 was considered statistically significant for all tests.

## 3 Results

### 3.1 Analysis of basic clinical data

This study enrolled a total of 81 healthy adult women, comprising 51 participants in the Yoga group and 30 in the non-Yoga group. Analysis of baseline demographic and clinical characteristics revealed no statistically significant differences (all p *>* 0.05) between the two groups regarding age, height, weight, or BMI, as detailed in Table 2.

**TABLE 2:** Baseline demographic and clinical characteristics.

Parameter	Yoga group (n = 51)	Non-Yoga group (n = 30)	*t*	*P*
Age (years)	41.59 ± 7.58	40.23 ± 6.50	−0.818	0.416
Height (cm)	159.71 ± 6.74	158.60 ± 6.08	−0.740	0.461
Weight (kg)	49.77 ± 2.34	49.79 ± 2.51	0.027	0.979
BMI (kg/m^2^)	19.36 ± 0.88	19.55 ± 1.25	0.802	0.425

BMI, body mass index.

### 3.2 Between-group and longitudinal comparisons of muscle thickness

To investigate changes in muscle thickness over time, between-group comparisons (Yoga vs. non-Yoga) at each time point and within-group longitudinal analyses were performed. The results demonstrated no statistically significant differences (all p *>* 0.05) in muscle thickness between the Yoga group and the non-Yoga group for the trapezius, long head of the biceps femoris, or semitendinosus muscles at any corresponding time point, as detailed in Table 3.

**TABLE 3:** Between-group and longitudinal comparisons of muscle thickness at different time points.

Time point/Group	Trapezius thickness (mm) Yoga group (n = 51)	Trapezius thickness (mm) Non-Yoga group (n = 30)	*t*_*1*_	*P*_*1*_	Biceps Femoris long head thickness (mm) Yoga group (n = 51)	Biceps Femoris long head thickness (mm) Non-yoga group (n = 30)	*t*_*2*_	*P*_*2*_	Semitendinosus thickness (mm) Yoga group (n = 51)	Semitendinosus thickness (mm) Non-yoga group (n = 30)	*t*_*3*_	*P*_*3*_
Pre-training	7.78 ± 1.65	7.26 ± 1.48	1.441	0.153	22.27 ± 3.72	21.26 ± 3.45	1.203	0.232	17.79 ± 2.55	16.89 ± 3.04	1.422	0.159
1-month post-training	7.79 ± 1.64	7.29 ± 1.50	1.371	0.174	22.23 ± 3.70	21.32 ± 3.44	1.092	0.278	17.79 ± 2.50	16.96 ± 2.99	1.346	0.182
3-month post-training	7.64 ± 1.65	7.34 ± 1.49	0.807	0.422	22.08 ± 3.69	21.33 ± 3.42	0.903	0.369	17.65 ± 2.53	17.00 ± 3.01	1.037	0.303
6-month post-training	7.48 ± 1.64	7.46 ± 1.49	0.054	0.957	21.91 ± 3.72	21.46 ± 3.44	0.540	0.591	17.47 ± 2.54	17.08 ± 3.01	0.615	0.540

*P*_*1*_: Comparison between Yoga and non-Yoga groups for trapezius thickness; *P*_*2*_: Comparison between Yoga and non-Yoga groups for biceps femoris long head thickness; *P*_*3*_: Comparison between Yoga and non-Yoga groups for semitendinosus thickness.

### 3.3 Relationship between different muscles and biomechanical parameters

SWE assessment of the trapezius and hamstring muscles—including the long head of the biceps femoris and the semitendinosus—in the Yoga group demonstrated significant differences in muscle elasticity (E_mean_) across various measurement postures and imaging orientations (all p < 0.05). These variations indicate that both measurement position and technique substantially influence the elasticity values obtained by SWE, as detailed in Table 4. The corresponding SWV data, showing consistent trends, are provided in Supplementary Table S1.

**TABLE 4:** Biomechanical characteristics across different muscles.

Muscle region	Posture/Measurement plane	E_mean_ (kPa)	*F*	*P*
Trapezius	Neutral/Transverse	20.62 ± 5.37	27.822	<0.001
	Neutral/Longitudinal	25.84 ± 6.51		
	Flexed/Transverse	29.47 ± 9.71		
	Flexed/Longitudinal	25.47 ± 6.61		
	Extended/Transverse	18.78 ± 6.77		
	Extended/Longitudinal	27.39 ± 6.06		
Long head of the biceps femoris	Neutral/Transverse	10.90 ± 3.54	39.448	<0.001
	Neutral/Longitudinal	13.42 ± 6.07		
	Flexed/Transverse	21.13 ± 8.60		
	Flexed/Longitudinal	18.74 ± 7.66		
Semitendinosus	Neutral/Transverse	10.85 ± 4.58	51.902	<0.001
	Neutral/Longitudinal	12.80 ± 6.14		
	Flexed/Transverse	23.26 ± 9.42		
	Flexed/Longitudinal	17.49 ± 6.58		

E_mean_ denotes mean Young’s modulus. Data are presented as mean ± standard deviation. *F*-values and *P*-values were derived from one-way analysis of variance (ANOVA). A *P*-value < 0.05 was considered statistically significant. Significant differences were observed for each muscle region across all evaluated measurement postures/techniques.

### 3.4 Correlation analysis between cumulative yoga training duration and biomechanical parameters across muscle regions

Analysis of the relationship between cumulative yoga training duration (defined as time since baseline) and muscle biomechanical parameters revealed weak but consistent inverse correlations between training duration and muscle elasticity (Emean) across multiple muscles and measurement conditions (Table 5). For the trapezius muscle, E_mean_ measured in the transverse plane during maximal forward head flexion showed a weak negative correlation with cumulative yoga training duration (r = −0.188, p < 0.05). In the long head of the biceps femoris, E_mean_ was inversely correlated with cumulative yoga training duration under three conditions: longitudinal plane in neutral knee position (r = −0.215, p < 0.05), transverse plane during 90° knee flexion (r = −0.225, p < 0.05), and longitudinal plane during 90° knee flexion (r = −0.293, p < 0.05). The semitendinosus muscle exhibited similar weak negative correlations in the longitudinal-neutral (r = −0.262, p < 0.05), transverse-flexed (r = −0.254, p < 0.05), and longitudinal-flexed (r = −0.242, p < 0.05) positions, as detailed in Table 5.

**TABLE 5:** Correlation analysis between cumulative yoga training duration and biomechanical parameters across muscle regions.

Muscle region	Posture/Measurement Plane	E_mean_ (kPa) *r*_*1*_	E_mean_ *P*_*1*_
Trapezius	Neutral/Transverse	−0.141	0.082
	Neutral/Longitudinal	−0.108	0.185
	Flexed/Transverse	−0.188	0.020*
	Flexed/Longitudinal	−0.126	0.121
	Extended/Transverse	−0.028	0.733
	Extended/Longitudinal	−0.004	0.964
Long head of the biceps femoris	Neutral/Transverse	−0.127	0.118
	Neutral/Longitudinal	−0.215	0.008*
	Flexed/Transverse	−0.225	0.005*
	Flexed/Longitudinal	−0.293	0.000*
Semitendinosus	Neutral/Transverse	−0.160	0.058
	Neutral/Longitudinal	−0.262	0.001*
	Flexed/Transverse	−0.254	0.002*
	Flexed/Longitudinal	−0.242	0.003*

E_mean_ denotes mean Young’s modulus. *r*_*1*_: Pearson’s correlation coefficient (*r*) between cumulative yoga training duration and E_mean_; *P*_*1*_: *P*-value for correlation between cumulative yoga training duration and E_mean._ A *P*-value < 0.05 was considered statistically significant, and significant correlations are marked with *.

Corresponding SWV data demonstrated comparable inverse relationships and are presented in Supplementary Table S2.

### 3.5 Changes in biomechanical parameters of the trapezius following exercise

**FIGURE 7:**
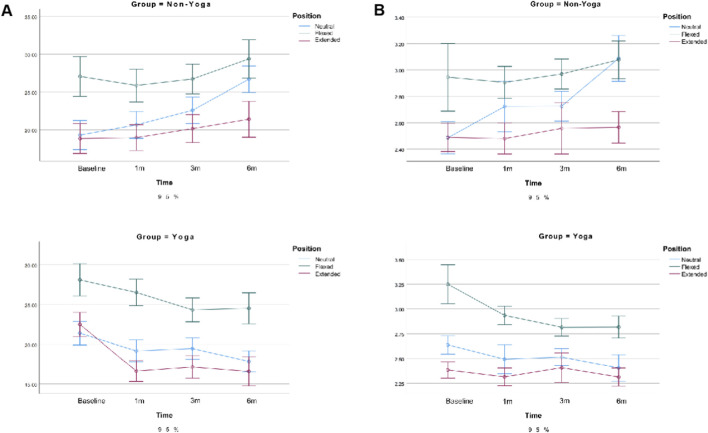
Changes in biomechanical parameters of the trapezius following exercise. Note: **(A)** Mean elasticity modulus (E_mean_) in the longitudinal section; **(B)** Mean elasticity modulus (E_mean_) in the transverse section. Error bars represent the 95% confidence interval (95% CI). p < 0.05 indicates significant differences between groups or across time points.

For E_mean_, both position (p < 0.001) and time (p = 0.014) showed significant main effects, along with a significant group × time interaction (p < 0.001). These findings indicate that the Yoga group exhibited a progressive decrease in muscle stiffness over time, whereas the Non-Yoga group remained relatively stable. The effect was more pronounced in the longitudinal section (ηp^2^ = 0.684) than in the transverse section (ηp^2^ = 0.042), suggesting higher sensitivity of longitudinal SWE measurements to muscle elasticity changes, as detailed in Figure 7.

Consistent trends were observed for SWV, which also showed time-dependent reductions in the Yoga group, particularly in neutral and flexed positions, further supporting the E_mean_ findings (see Supplementary Figure S1).

### 3.6 Changes in biomechanical parameters of the hamstring muscles following exercise

For the biceps femoris long head, significant main effects were observed for position (longitudinal: F (1, 79) = 233.62, p < 0.001, ηp^2^ = 0.747; transverse: F (1, 79) = 182.80, p < 0.001, ηp^2^ = 0.698) and time (longitudinal: F (2.66, 210.24) = 6.57, p < 0.001, ηp^2^ = 0.077; transverse: F (2.42, 191.04) = 7.52, p < 0.001, ηp^2^ = 0.087), along with a significant group × time interaction (longitudinal: F (2.66, 210.24) = 12.11, p < 0.001, ηp^2^ = 0.133; transverse: F (2.42, 191.04) = 9.19, p < 0.001, ηp^2^ = 0.104). In addition, a significant position × time interaction was observed (F (2.66, 209.94) = 7.10, p < 0.001, ηp^2^ = 0.083).

The Yoga group showed a gradual decrease in E_mean_ across time, especially in neutral and flexed positions, while the Non-Yoga group remained relatively stable. The longitudinal section demonstrated higher sensitivity (ηp^2^ = 0.75 vs. 0.70).

For the semitendinosus, position (F (1, 79) = 210.12, p < 0.001, ηp^2^ = 0.727; F (1, 79) = 116.94, p < 0.001, ηp^2^ = 0.597), time (F (2.05, 161.87) = 4.19, p = 0.007, ηp^2^ = 0.050; F (2.47, 195.00) = 3.37, p = 0.027, ηp^2^ = 0.041), and group × time interaction (F (2.05, 161.87) = 9.93, p < 0.001, ηp^2^ = 0.113; F (2.47, 195.00) = 5.44, p = 0.003, ηp^2^ = 0.064) were all significant in the longitudinal and transverse sections, respectively.

**FIGURE 8:**
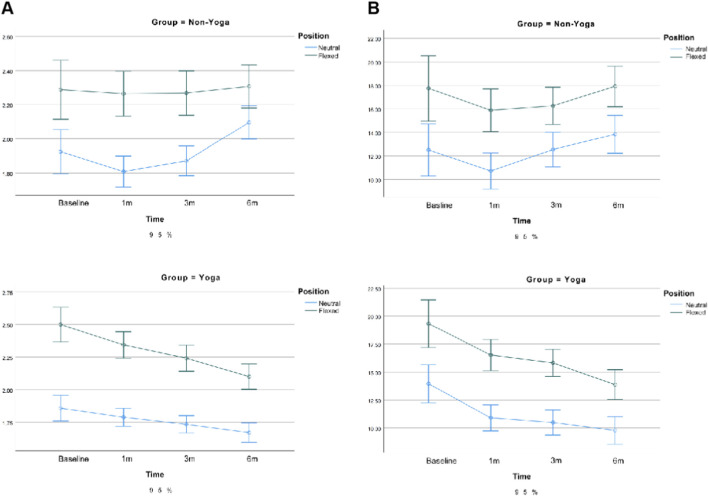
Changes in biomechanical parameters of the long head of the biceps femoris following exercise. Note: **(A)** Mean elasticity modulus (E_mean_) in the longitudinal section; **(B)** Mean elasticity modulus (E_mean_) in the transverse section. Error bars represent the 95% confidence interval (95% CI). p < 0.05 denotes statistically significant differences.

**FIGURE 9:**
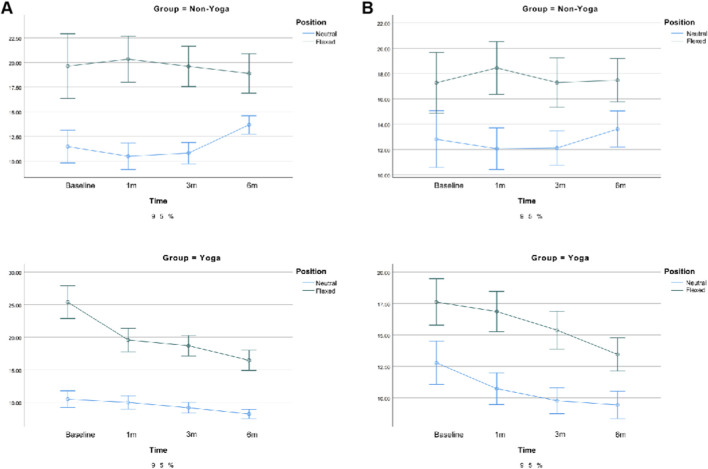
Changes in biomechanical parameters of the semitendinosus following exercise. Note: **(A)** Mean elasticity modulus (E_mean_) in the longitudinal section; **(B)** Mean elasticity modulus (E_mean_) in the transverse section. Error bars represent the 95% confidence interval (95% CI). p < 0.05 indicates significant effects of group or time.

E_mean_ decreased progressively in the Yoga group, particularly at neutral and flexed positions, indicating reduced muscle stiffness and improved elasticity. The longitudinal section again showed greater responsiveness (ηp^2^ = 0.73 vs. 0.60). See Figures 8, 9. Similar trends were detected for SWV, reinforcing the observed improvements in tissue elasticity (see Supplementary Figures S2, S3).

## 4 Discussion

This study found that participation in a 6-month Yoga training program was associated with significant reductions in passive muscle stiffness in both the trapezius and hamstring muscles, as evidenced by decreased shear-wave elastography-derived measures of mean elasticity (E_mean_) and shear-wave velocity (SWV) (SWE) (Liu et al., 2024). Stiffness declines became apparent after 3 months and were most pronounced at 6 months, suggesting a progressive pattern of change in muscle elastic properties over time. Notably, these changes occurred without any increase in muscle thickness, indicating that the observed adaptations were primarily functional (viscoelastic) rather than structural (i.e., hypertrophy). Increased soft-tissue elasticity may be associated with enhanced flexibility and joint range of motion, potentially contributing to better postural stability and reduced injury risk in Yoga practitioners. This interpretation is consistent with prior evidence that sustained stretching regimens are associated with decreases in musculotendinous stiffness. A recent meta-analysis confirmed that 3–12 weeks of static stretching can produce a moderate reduction in muscle stiffness compared to no exercise (effect size ≈ -0.75) (Takeuchi et al., 2023). Likewise, acute stretching has been shown to transiently lower muscle stiffness in young adults (Yoshimura et al., 2025), highlighting the capacity of flexibility-focused exercise to improve muscle compliance. Thus, the observed decrease in SWE-derived Emean/SWV after Yoga likely reflects favorable changes in the muscles’ viscoelastic properties, enhancing their extensibility and functional length without necessitating morphological changes.

The three-way repeated-measures ANOVA revealed significant group × time interactions (p < 0.001), indicating that temporal changes in muscle stiffness differed over time between the Yoga and non-Yoga groups. In addition, muscle stiffness varied across measurement positions (p < 0.001), and consistent differences were observed between longitudinal and transverse measurement planes when analyses were performed separately. In particular, transverse-plane measurements during maximal forward head flexion (trapezius) and 90° knee flexion (hamstrings) demonstrated the highest reproducibility (Stiver et al., 2023). These results highlight the utility of SWE as a reliable and quantitative technique for detecting subtle, yoga-associated changes in muscle mechanical properties, providing insight into neuromuscular adaptations that may not be fully captured through conventional morphological or strength assessments (Chalchat et al., 2022; Licen and Kozinc, 2022; Stiver et al., 2023). Previous studies have similarly reported good repeatability of SWE measures in skeletal muscle (e.g., trapezius stiffness; (Kozinc and Sarabon, 2020)) and have recommended reporting shear-wave velocity directly to minimize error (Stiver et al., 2023).

From a functional perspective, the reduction in muscle stiffness observed in association with Yoga practice appears beneficial for activities emphasizing flexibility, control, and stretch tolerance, but it is not universally advantageous across all contexts. In sports requiring explosive power or elastic recoil, higher muscle-tendon stiffness can enhance force transmission and performance. For example, athletes with greater lower-limb stiffness have demonstrated superior jump and sprint outcomes (Kalkhoven and Watsford, 2018). Conversely, in endurance or high-volume loading scenarios, transient decreases in stiffness often accompany fatigue and tissue remodeling; post-exercise studies have observed stiffness reductions as part of the recovery process (e.g., after competitive matches, (Goulart et al., 2022)). It is important to note that a reduction in SWE-derived average stiffness (i.e., lower E_mean_/SWVmean) does not universally indicate superior muscle function across all sports. In explosive or jump-based athletics, higher stiffness may benefit force transmission and elastic recoil; for instance, Kalkhoven et al. observed that greater lower limb stiffness correlated with better performance in power tasks (Kalkhoven and Watsford, 2018). Conversely, in flexibility- and control-focused training such as Yoga, lower muscle stiffness may better support joint mobility, stretch tolerance, and injury resilience. Moreover, in endurance or high-volume loading, reductions in stiffness may also reflect remodeling, microdamage recovery, or fatigue adaptation (Thomas et al., 2022).

In our study, we interpret the observed decrease in E_mean_/SWVmean as a favorable adaptation associated with the demands of sustained flexibility and postural control in Yoga, and we do not extend this interpretation to strength/power sports. Within this context, we interpret the Yoga-induced decrease in Emean/SWV as a favorable adaptation aligned with the demands of sustained stretching and postural control in Yoga. The softer, more pliable muscle state likely facilitates improved joint mobility and may reduce strain on tendons and connective tissues, potentially contributing to injury prevention and better neuromuscular coordination during daily activities. We caution that this adaptation is specific to a flexibility-focused regimen and should not be generalized to all athletic pursuits without careful consideration of sport-specific requirements.

Broader implications of changes in muscle pliability are relevant for neuromuscular health, rehabilitation, and wellness. Notably, changes in muscle elasticity have been reported across multiple interventions: a 2024 randomized trial found that 5 weeks of therapeutic massage was associated with reduced upper trapezius stiffness, underscoring that muscle pliability can be enhanced via different modalities (Jelen et al., 2024). Consistent with our findings, Yoga-based interventions have been shown to enhance neuromuscular coordination and functional balance in diverse populations. An occupational therapy review reported that Yoga was associated with postural control, flexibility, and balance in adults at risk of falls (including older individuals and patients with neurological conditions), with these effects being attributed to enhanced neuromuscular synergy (Green et al., 2019). Likewise, a recent meta-analysis in knee osteoarthritis patients concluded that Yoga training induces more coordinated muscle activation around the knee, leading to better alignment and joint stability (Lu et al., 2024). Such changes in muscle elasticity and control have practical applications in clinical rehabilitation and sports medicine. Incorporating Yoga or similar stretching programs into rehabilitation may help increase muscle flexibility, reduce stiffness-related pain, and restore range of motion, while athletes could use Yoga during recovery phases to maintain muscle suppleness and prevent injury. Beyond clinical and sports settings, workplace wellness programs may also benefit: a randomized trial showed that just 10 min of daily office Yoga for 1 month was associated with reduced neck and back musculoskeletal discomfort in sedentary workers compared to controls (Garcia et al., 2023). Finally, the use of SWE in this study illustrates the potential advantage of objective monitoring of muscle biomechanical properties. Ultrasound elastography provides a reproducible, real-time measure of tissue stiffness changes, allowing clinicians and researchers to quantitatively track improvements in muscle quality over time and to tailor interventions for optimal neuromuscular outcomes. This pattern aligns with findings from a recent randomized controlled trial involving individuals with Parkinson’s disease, where an 8-week Yoga intervention led to a significant decrease in medial gastrocnemius stiffness (SWV) compared to a power-training group (Ni et al., 2016). Such longitudinal evidence suggests that SWE can sensitively capture functional improvements resulting from Yoga, providing objective monitoring of muscle quality gains over time, which is an effect absent whereas such changes were not observed in non-Yoga cohorts (Romer et al., 2022).

The observed changes in trapezius and hamstring muscle elasticity may plausibly be related to the inclusion of sustained, low-load static asanas in the yoga program. Postures such as Adho Mukha Svanasana, Uttanasana, Paschimottanasana, and Setu Bandha Sarvangasana impose prolonged tensile loading on the posterior chain and cervical-shoulder musculature, which may be associated with viscoelastic adaptations in muscle tissue over time. In particular, forward-bending postures (e.g., Uttanasana and Paschimottanasana) involve sustained lengthening of the hamstring muscles, whereas postures requiring active shoulder stabilization and cervical positioning (e.g., Adho Mukha Svanasana) may influence the mechanical properties of the upper trapezius. When practiced repeatedly over an extended period, such low-intensity, static stretching stimuli may be associated with reductions in passive muscle stiffness and corresponding changes in elastic properties.

This study has several limitations that should be acknowledged. First, menstrual cycle phase was not controlled or documented, although hormonal fluctuations are known to influence muscle stiffness in women, which may have contributed to inter-individual variability in shear-wave elastography measurements. Second, group allocation was based on participants’ willingness to engage in yoga rather than randomization, introducing potential selection bias and residual confounding. Although a standardized yoga protocol was implemented, future randomized controlled trials with fully supervised and objectively monitored training load are warranted. Individuals choosing yoga practice may have differed from controls in motivation, lifestyle, or baseline physical activity. Moreover, physical activity outside the yoga sessions (e.g., other exercise, occupational activity, and daily activity levels) was not systematically monitored or controlled, and its potential contribution to the observed changes cannot be excluded. Therefore, the findings should be interpreted as associational rather than causal. In addition, due to practical recruitment constraints, particularly the low participation rate of males in yoga practice, the cohort consisted exclusively of female participants, limiting the generalizability of the findings across genders. The relatively modest sample size further restricts broader applicability. Although a standardized training structure was implemented, individual training intensity and posture-specific loading were not quantitatively assessed, which may limit detailed comparisons with other intervention protocols. Furthermore, a formal between-session test-retest reliability assessment was not conducted, and thus between-day ICC values are unavailable.

Future studies should employ larger, multi-center cohorts with balanced sex representation, incorporate detailed monitoring of overall physical activity and training load, and include repeated measurements to assess long-term reproducibility. Expanding the range of examined muscle groups and systematically comparing different yoga styles may provide a more comprehensive understanding of yoga-related biomechanical adaptations. Finally, mechanistic studies exploring neuromuscular and structural pathways underlying yoga-associated changes in muscle biomechanics are warranted.

## 5 Conclusion

The findings suggest that Yoga practice may be associated with favorable changes in muscle mechanical properties in healthy adult women. However, given the self-selected group allocation and the lack of control for physical activity outside yoga, these results should be interpreted as associational and hypothesis-generating. Larger studies with improved control of confounding factors are warranted to confirm these findings.

## Data Availability

The original contributions presented in the study are included in the article/Supplementary Material, further inquiries can be directed to the corresponding author.

## Supplementary material

SUPPLEMENTARY FIGURE S1 Changes in biomechanical parameters of the trapezius following exercise (SWV).

SUPPLEMENTARY FIGURE S2 Changes in biomechanical parameters of the long head of the biceps femoris following exercise (SWV).

SUPPLEMENTARY FIGURE S3 Changes in biomechanical parameters of the semitendinosus following exercise (SWV).
