# Supplementary material for: Yoga training enhances elastic biomechanics of trapezius and hamstrings: a quantitative SWE assessment
Source: Front Physiol. 2026 Jan 13;16:1671051. doi: 10.3389/fphys.2025.1671051 (PMC12834789; doi:10.3389/fphys.2025.1671051)
Supplement: Supplementary file 1 [file Table1.docx]

Table S1 Biomechanical characteristics across different muscles

| Muscle region | Posture/Measurement Plane | SWV_mean_ (cm/s) | *F* | *P* |
| --- | --- | --- | --- | --- |
| **Trapezius** | Neutral/Transverse | 2.58 ± 0.34 | 14.956 | < 0.001 |
|  | Neutral/Longitudinal | 3.04 ± 1.01 |  |  |
|  | Flexed/Transverse | 3.14 ± 0.72 |  |  |
|  | Flexed/Longitudinal | 2.98 ± 0.82 |  |  |
|  | Extended/Transverse | 2.42 ± 0.30 |  |  |
|  | Extended/Longitudinal | 3.05 ± 0.63 |  |  |
| Long head of the biceps femoris | Neutral/Transverse | 1.88 ± 0.35 | 26.684 | < 0.001 |
|  | Neutral/Longitudinal | 2.04 ± 0.46 |  |  |
|  | Flexed/Transverse | 2.42 ± 0.49 |  |  |
|  | Flexed/Longitudinal | 2.39 ± 0.53 |  |  |
| Semitendinosus | Neutral/Transverse | 1.84 ± 0.35 | 21.035 | < 0.001 |
|  | Neutral/Longitudinal | 2.03 ± 0.62 |  |  |
|  | Flexed/Transverse | 2.61 ± 0.85 |  |  |
|  | Flexed/Longitudinal | 2.34 ± 0.74 |  |  |

Table S2 Correlation analysis between exercise duration and biomechanical parameters across muscle regions

| Muscle region | Posture/Measurement Plane | SWV_mean_ (m/s) *r_2_* | SWV_mean_ *P_2_* |
| --- | --- | --- | --- |
| **Trapezius** | Neutral/Transverse | -0.034 | 0.676 |
|  | Neutral/Longitudinal | -0.108 | 0.183 |
|  | Flexed/Transverse | -0.199 | 0.013* |
|  | Flexed/Longitudinal | -0.126 | 0.120 |
|  | Extended/Transverse | -0.086 | 0.288 |
|  | Extended/Longitudinal | -0.002 | 0.984 |
| Long head of the biceps femoris | Neutral/Transverse | -0.107 | 0.189 |
|  | Neutral/Longitudinal | -0.217 | 0.007* |
|  | Flexed/Transverse | -0.196 | 0.015* |
|  | Flexed/Longitudinal | -0.263 | 0.001* |
| Semitendinosus | Neutral/Transverse | -0.140 | 0.084 |
|  | Neutral/Longitudinal | -0.252 | 0.002* |
|  | Flexed/Transverse | -0.216 | 0.007* |
|  | Flexed/Longitudinal | -0.224 | 0.005* |
