# Supplementary figures and images for: Yoga training enhances elastic biomechanics of trapezius and hamstrings: a quantitative SWE assessment
Source: Front Physiol. 2026 Jan 13;16:1671051. doi: 10.3389/fphys.2025.1671051 (PMC12834789; doi:10.3389/fphys.2025.1671051)

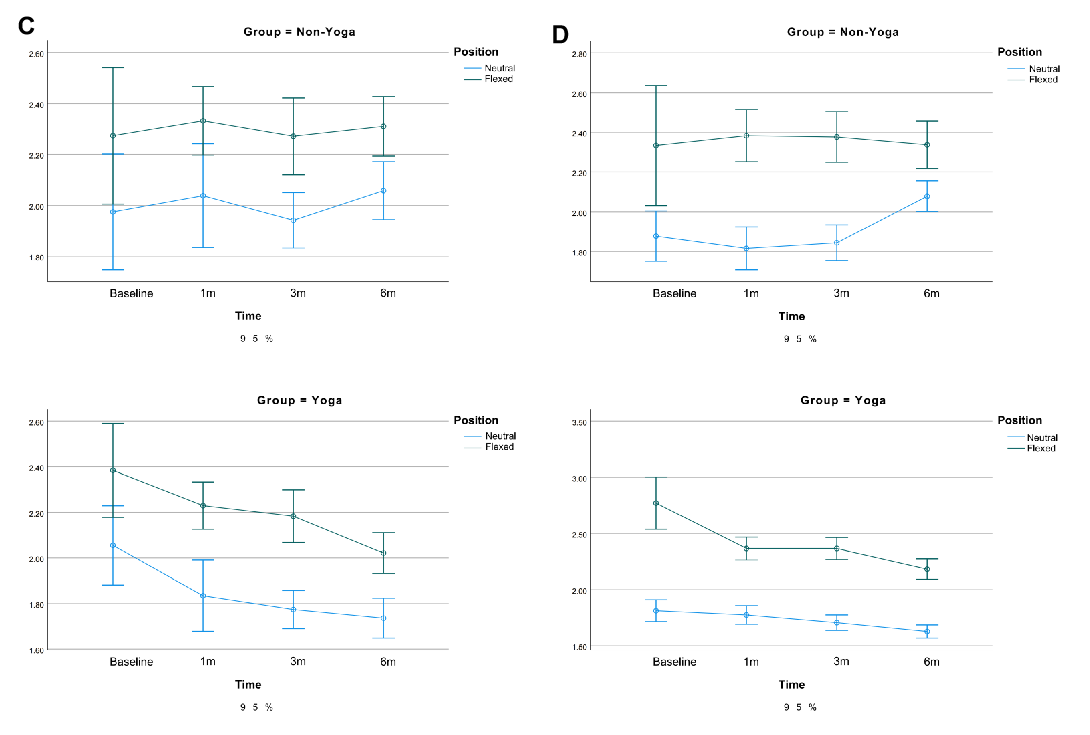

Supplement: Supplementary file 2 [file Image3.tif]

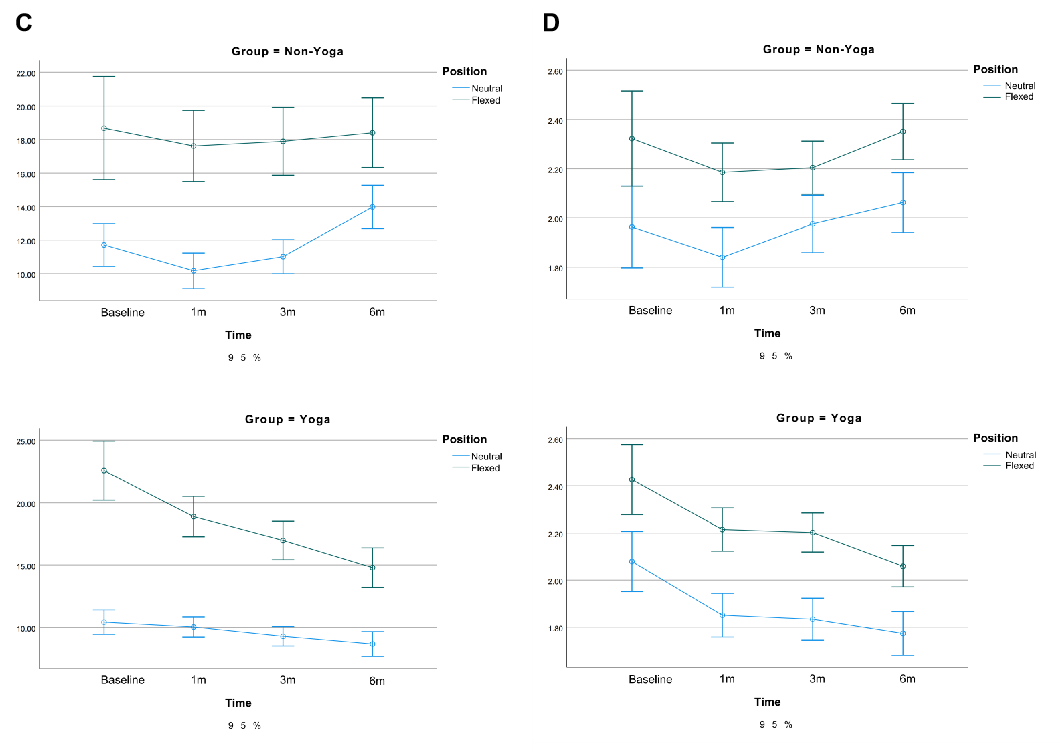

Supplement: Supplementary file 3 [file Image2.tif]

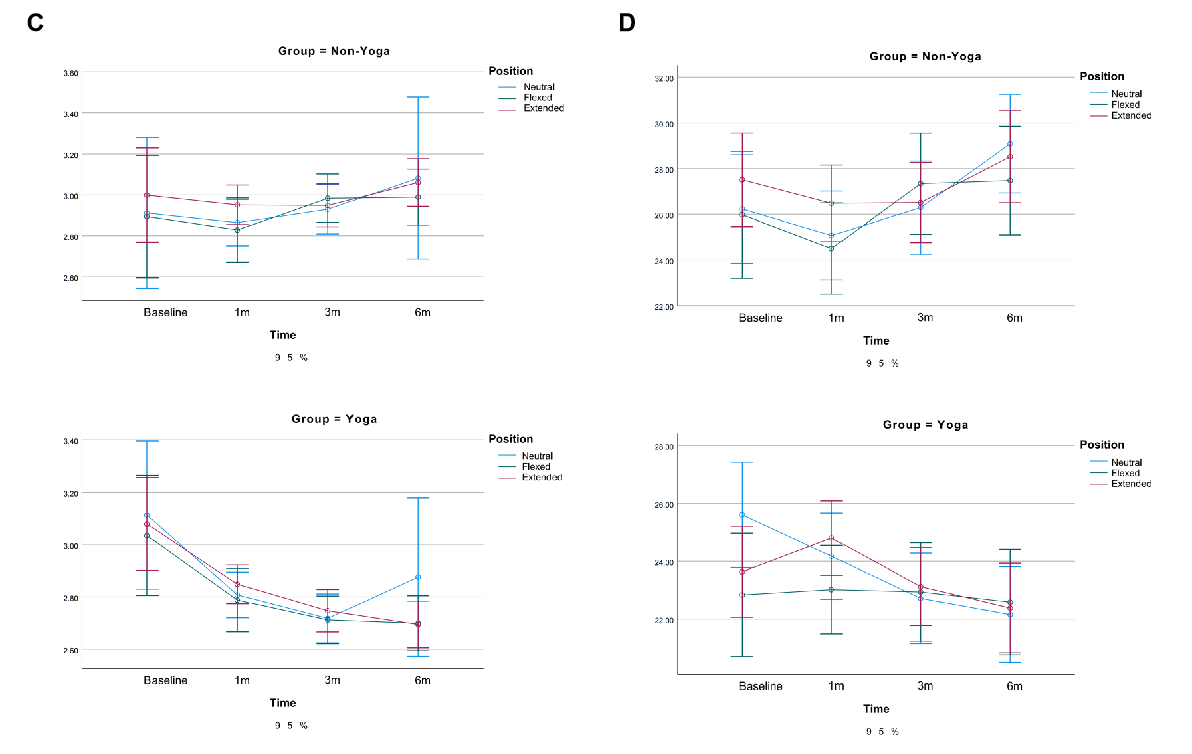

Supplement: Supplementary file 4 [file Image1.png]
